# West Nile Virus in Golden Eagles, Spain, 2007

**DOI:** 10.3201/eid1409.080190

**Published:** 2008-09

**Authors:** Miguel Angel Jiménez-Clavero, Elena Sotelo, Jovita Fernandez-Pinero, Francisco Llorente, Juan Manuel Blanco, Julia Rodriguez-Ramos, Elisa Perez-Ramirez, Ursula Höfle

**Affiliations:** Centro de Investigación en Sanidad Animal, Valdeolmos, Spain (M.A. Jiménez-Clavero, E. Sotelo, J. Fernandez-Pinero, F. Llorente); Centro de Estudios de Rapaces Ibéricas, Sevilleja de la Jara, Spain (J.M. Blanco, J. Rodriguez-Ramos, U. Höfle); Instituto de Investigación en Recursos Cinegéticos, Ciudad Real, Spain (E. Perez-Ramirez, U. Höfle)

**Keywords:** West Nile virus, wild birds, golden eagle, *Aquila chrysaetos*, virus isolation, Spain, letter

**To the Editor:** Although West Nile virus (WNV) has not been isolated in Spain, several recent studies provide evidence for its circulation in this country (*1*–*5*). We report isolation of WNV in Spain from 2 golden eagles (*Aquila chrysaetos*).

A captive-bred 2-year-old male golden eagle (GE-1) was released into the wild in central Spain. The bird’s location was monitored daily by telemetry, and it remained within a radius of 100 km from its original release point. On September 15, 2007 (1 month after release), it was found moribund and was moved to a rehabilitation and captive breeding center for endangered raptors. Upon admission, the bird was in fair condition but debilitated and aggressive. It then became increasingly disorientated, showed a head tilt, and died 5 days after admission, despite intensive supportive care and treatment for secondary infections.

Eleven days after admission of GE-1, an adult male golden eagle (GE-2) and an adult female Bonelli’s eagle (*Hieraaetus fasciatus* [BE-1]) living in pairs (with a golden eagle and a Bonelli’s eagle, respectively) in enclosures were found disorientated, debilitated, and with impaired vision. Both birds where placed in isolation and received intensive supportive care; they slowly recovered. The respective pair of each bird (GE-3 and BE-2, respectively) remained asymptomatic. A magpie (MP-1) that had entered the golden eagle enclosure 5 days before admission of GE-1 was also placed in isolation, but remained healthy. After necropsy of GE-1, tissue samples (brain, kidney, and spleen) from this bird and oropharyngeal swabs from GE-2, BE-1, and MP-1 (obtained at day 11 after admission of GE-1) were subjected to virologic analysis.

Avian influenza and Newcastle disease were excluded by reverse transcription–PCR (RT-PCR) (*6*,*7*) of oropharyngeal and cloacal swabs from GE-1, GE-2, BE-1, and MP-1. Real-time RT-PCR specific for WNV (*8*) was conducted with brain, kidney, and spleen tissue homogenates from GE-1 and oropharyngeal swabs from GE-2, BE-1, and MP-1. All samples except that from MP-1 yielded specific WNV genome amplification products, which were confirmed after amplification and sequencing by using a previously described method (*9*).

Serum samples from clinically affected eagles (GE-1, GE-2, and BE-1), the magpie (MP-1), and the healthy Bonelli’s eagle (BE-2) contained WNV-neutralizing antibodies detected by a virus neutralization test performed as described (*4*,*5*). A serum sample from GE-3 (asymptomatic) remained negative up to 74 days after admission of GE-1. Specificity of the neutralization test was assessed by titration in parallel against a second, cross-reacting flavivirus (Usutu virus). Results showed that the highest titers were always obtained against homologous virus (WNV).

Virus isolation was conducted by placing filter-sterilized, clarified tissue homogenates (brain, kidney and spleen) from GE-1 and oropharyngeal swab eluate from GE-2 onto monolayers of BSR (baby hamster kidney) cells and Vero cells. The remaining 2 samples (oropharyngeal swabs from BE-1 and MP-1) were negative for virus. Isolates were identified by using real-time and conventional RT-PCR (*8*,*9*). WNV-specific cDNAs from the nonstructural protein 5–coding region of the genome (171 nt) were amplified by RT-PCR (*9*) from brain tissue of GE-1 (sample GE-1b), oropharyngeal swab of BE-1 (sample BE-1o), and first-passage infection supernatant of oropharyngeal swab from GE-2 (sample GE-2o). These samples were subjected to molecular analysis. Nucleotide sequences from the 3 samples were identical, except at 1 nt position in BE-1o (GenBank accession nos. EU486169 for GE-1b, EU486170 for GE-2o, and EU486171 for BE-1o). Phylogenetic analysis matched these isolates most closely with recent western Mediterranean WNV isolates within lineage 1a (Figure).

**Figure:**
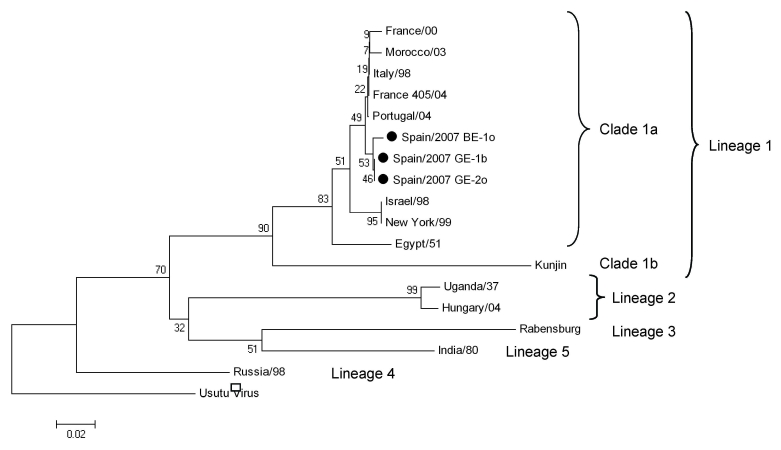
Phylogenetic tree of 18 partial nonstructural protein 5 West Nile virus nucleotide sequences (171 nt for each isolate, except 126 nt available for the Portugal/04 isolate) constructed with MEGA version 4 software (www.megasoftware.net). The optimal tree was inferred by using the neighbor-joining method. The percentage of successful bootstrap replicates (N = 1,000) is indicated at nodes. Evolutionary distances were computed with the Kimura 2-parameter method (with gamma correction). All positions containing alignment gaps and missing data were eliminated only in pairwise sequence comparisons. Branch lengths are proportional to the number of nucleotide changes (genetic distances). Scale bar shows number of base substitutions per site. Isolates sequenced in this study are indicated by solid circles. GenBank accession nos. are as follows: France/00 (AY268132), Morocco/03 (AY701413), Italy/98 (AF404757), France 405/04 (DQ786572), Portugal/04 (AJ965630), Israel/98 (AF481864), New York/99 (DQ211652), Egypt/51 (AF260968), Kunjin (D00246), Uganda/37 (M12294), Hungary/04 (DQ116961), Rabensburg (AY765264), India/80 (DQ256376), Russia/98 (AY277251), and Usutu virus (NC_006551) (outgroup).

WNV was detected in 3 eagles of 2 species. The birds with the index and secondary cases had no direct contact. Transmission could have occurred through mosquito bites. The 2-year-old golden eagle died as a result of infection, and the 2 remaining infected eagles recovered. The 3 ill birds were potentially more susceptible because of stress (GE-1) or age (GE-2 and BE-1 were older birds). Serologic analysis detected WNV-specific antibodies in the affected birds and some contacts. Nucleotide sequence analysis showed high genetic identity among these new isolates, which cluster within lineage 1a of WNV.

Although information on WNV in Spain is scarce, its detection and relationship to the death of a raptor in the wild are of concern because many species of eagles, including the Spanish imperial eagle (*A*. *adalberti*), are endangered species. We recently found evidence of WNV infection in several Spanish imperial eagles sampled during 2001–2005 (*5*). Studies are ongoing to further characterize genetic and biologic properties of the new WNV isolates described to identify their genetic relationships with other WNV strains and to clarify the epidemiology of WNV in the study region.
